# A three-year data set of gaseous field emissions from crop sequence at three sites in Germany

**DOI:** 10.1038/s41597-022-01549-2

**Published:** 2022-07-16

**Authors:** Janine Mallast, Heinz Stichnothe, Thomas Kreuter, Enrico Thiel, Claudia Pommer, Johannes Döhler, Florian Eissner, Insa Kühling, Jan Rücknagel, Henning Pamperin, Jürgen Augustin, Mathias Hoffmann, Anja Simon, Kurt-Jürgen Hülsbergen, Franz-Xaver Maidl, Nadine Tauchnitz, Joachim Bischoff, Falk Böttcher

**Affiliations:** 1 Thuenen Institute of Agricultural Technology, Braunschweig, Germany; 2 SKW Stickstoffwerke Piesteritz GmbH (SKWP), Experimental site Cunnersdorf, Leipzig, Germany; 3 Institute of Agricultural and Nutritional Science, Martin-Luther-University Halle-Wittenberg, Wittenberg, Germany; 4 Institute of Crop Science and Plant Breeding, Christian-Albrechts-University, Kiel, Germany; 5 Leibniz Centre for Agricultural Landscape Research (ZALF), Muencheberg, Germany; 6 Chair of Organic Agriculture and Agronomy, Technical University of Munich (TUM), Munich, Germany; 7 State Institute of Agriculture and Horticulture Saxony-Anhalt (LLG), Bernburg-Strenzfeld, Bernburg, Germany; 8 German Weather Service, Department Agrometeorology, Branch office Leipzig, Leipzig, Germany

**Keywords:** Agriculture, Environmental impact

## Abstract

The purpose of the StaPlaRes project was to evaluate two innovative techniques of urea fertiliser application and to quantify greenhouse gas (GHG) emissions. All GHG emissions, as well as other gaseous emissions, agronomic and environmental variables were collected for three years (2016/2017–2018/2019) at three experimental field sites in Germany. All management activities were consistently documented. Multi-variable data sets of gas fluxes (N_2_O and NH_3_), crop parameters (grain and straw yield, N content, etc.), soil characteristics (NH_4_-N, NO_3_-N, etc.), continuously recorded meteorological variables (air and soil temperatures, radiation, precipitation, etc.), management activities (sowing, harvest, soil tillage, fertilization, etc.), were documented and metadata (methods, further information about variables, etc.) described. Additionally, process-related tests were carried out using lab (N_2_ emissions), pot and lysimeter experiments (nitrate leaching). In total, 2.5 million records have been stored in a Microsoft Access database (StaPlaRes-DB-Thuenen). The database is freely available for (re)use by others (scientists, stakeholders, etc.) on the publication server and data repository OpenAgrar for meta-analyses, process modelling and other environmental studies.

## Abstract (summary)

**Table:** 

Measurement(s)	air temperature • air humidity • air pressure • global radiation • precipitation • Grain yield • nitrous oxide concentrations (gas field measurements) • emissions of NH3 • soil moisture • ammonium in soil • nitrate in soil
Technology Type(s)	weather station • Weighing • static closed chamber technique • method of calibrated Passive sampling • SENTEK sensor • Extraction with 0.0125 M CaCl2 solution or 0.1 M KCl (VDLUFA method handbook I A 6.1.4.1)
Factor Type(s)	Fertilizer
Sample Characteristic - Environment	gaseous field emissions
Sample Characteristic - Location	Germany

## Background & Summary

Worldwide use of urea has increased more than 100-fold in the past four decades and now constitutes more than 50% of global nitrogenous fertiliser usage^1^. The global urea market demand reached a volume of nearly 187.8 million metric tons in 2020. From 2021 to 2026 the demand is expected to grow by 2% annually^2^. A large percentage of urea-N used for food production is lost to the environment in many different forms, including NH_3_, N_2_O and N_2_ emissions^3–7^. Nitrous oxide contributes to both, the greenhouse effect^8^ and stratospheric ozone depletion^9,10^. More than half of the entire anthropogenic N_2_O emission originates from agricultural soils^11^. Ammonia (NH_3_) emission from agricultural sources significantly contributes to air pollution, soil acidification, water eutrophication, biodiversity loss, and declining human health^12^. There are numerous options for reducing NH_3_ emission from urea-fertilised agricultural systems. Inhibitors, for example, are a promising tool for N_2_O and NH_3_ mitigation. However, the effectiveness is highly variable and some measures depend widely on site-specific conditions such as weather, soil properties and management practices^13^. Moreover, there can be trade-offs such as simultaneous NH_3_ reduction and N_2_O increase.

In order to combine NH_3_ and N_2_O measurements, yield analyses, and soil sampling, a three-part experimental setup (see detailed explanation in Methods) was designed at three experimental field sites in Germany.

During the project period (autumn 2016 to autumn 2019) the weather was exceptionally warm and dry. The average annual temperature was 3 K higher compared to the long-term annual temperature at all experimental sites. A high deficit in annual precipitation occurred also at all sites, which was mainly caused by the lack of precipitation in spring. The second and third investigation years were significantly drier than the first one (see Table 1). These weather conditions were in line with the increasingly frequent droughts in Central Europe over the past 14 years^14^.

**Table 1:** Meteorological characteristics of the experimental field sites.

Site/Long-term annual mean and sum	Year	Annual temperature (°C)	Deviation from LTAM (K)	Annual precipitation sum (mm)	Deviation from LTAS (mm)
Bernburg (BER) (10.1 °C, 515 mm)^*^	2017	9.8	+0.3	513	−2
	2018	11.4	+1.3	372	−143
	2019	12.0	+1.9	437	−78
Cunnersdorf (CUN) (9.6 °C, 602 mm)^#^	2017	10.3	+0.7	778	+176
	2018	11.2	+1.6	397	−205
	2019	12.6	+3.0	478	−124
Roggenstein (ROG) (8.7 °C, 914 mm)^$^	2017	9.1	+0.4	810	−104
	2018	10.1	+1.4	868	−46
	2019	11.1	+2.4	600^&^	not evaluable

long-term annual mean of air temperature (LTAM) and long-term annual sum of precipitation (LTAS): *1990–2019; ^#^1989–2018; ^$^1995–2016.

^&^Sum from January until mid-October 2019.

Overall, the site-specific emission factors (EFs) for N_2_O range from 0% to 0.54%. These EFs are lower than the EFs according to The Global Nitrous Oxide calculator (GNOC)^15,16^ at the sites (EF 0.67–0.77%) and significantly lower than the default value of 1.0% according to the IPCC Refinement^17^. The trial-specific EFs fall within the lower uncertainty range of the aggregate N_2_ON- EF according to IPCC Refinement, which is reported to be 0.1–1.8%.

The NH_3_-N emission factor average over the three trial years and the three crops for the benchmark treatment “surface” is highest in Cunnersdorf (0.032 kg NH_3_-N kg N^−1^) and lowest in Roggenstein (0.012 kg NH_3_-N kg N^−1^). Overall, the specific measured NH_3_-N emission factors at all experimental sites during the project period from 2016 to 2019 are significantly below the default values for urea according to EMEP/EEA^18^ or Rösemann, *et al*.^19^ (0.142 kg NH_3_-N kg N^−1^, uncertainty range 0.03–0.43 kg NH_3_-N kg N^−1^).

We introduce multi-variable datasets of GHG emissions as well as other gaseous emissions and agronomic variables. All variables were collected for three years (2016/2017–2018/2019) at three experimental field sites in Germany. In total 2.5 million records have been stored and archived in the database StaPlaRes-DB-Thuenen to quantify and to evaluate GHG for winter oilseed rape, winter wheat and winter barley. The database is publicly available at the OpenAgrar repository^20^ (10.3220/DATA20220119144442). A virtual final event was organised, where project results were presented. The final report^21^, posters and presentations of the event are available at the website^22^. Some project results have already been published^23–25^.

## Methods

### Study field sites

The StaPlaRes project consists of three sites spread across Germany. The main soil characteristics of each field site are shown in Table 2.

**Table 2:** Soil characteristics of the experimental field sites.

Site	Height above nn (m)	Latitude	Longitude	Soil type (FAO Classification)^46^	Soil texture (%)			pH	Corg (%)
					Sand	silt	clay		
BER	80	51.816°	11.70°	Haplic Chernosem	9	69	22	7.1	1.4
CUN	140	51.210°	12.33°	Stagnosol	44	45	11	6.4	0.96
ROG	520	48.183°	11.33°	Luvisol	33	51	16	6.0	1.22

**Fig. 1:**
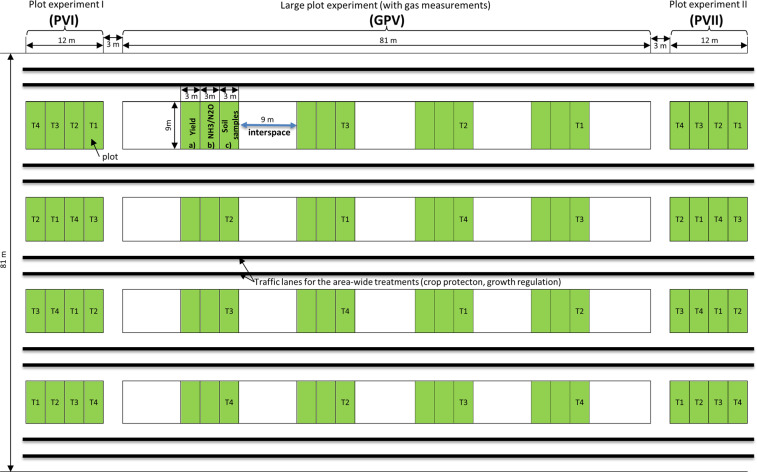
Spatial scheme of the experimental design of the StaPlaRes project.

The project was established in late summer 2016 to evaluate two innovative technologies of urea fertilization. At all field sites, oat (*Avena sativa L*.) was cultivated as the preceding crop to achieve comparable conditions. The experiment at each field site was designed as a uniform field trial with an identical crop sequence consisting of winter oilseed rape (*Brassica napus L.; short: OSR*) – winter wheat (*Triticum aestivum L.; short: WW*) – winter barley (*Hordeum vulgare L.; short: WB*). The experiment was divided in three plot experiments: plot experiment I (short: PVI), large plot experiment (short: GPV) and plot experiment II (short: PVII) (see Fig. 1). Randomization of the test elements was performed in each of the three plot-trials through Latin squares (n = 4). One crop was grown at one plot each year (see Table 3).

**Table 3:** Cultivation plan of the StaPlaRes project.

Experiment	2016/17	2017/18	2018/19
PVI	Winter barley	Winter oilseed rape	Winter wheat
GPV	Winter oilseed rape	Winter wheat	Winter barley
PVII	Winter wheat	Winter barley	Winter oilseed rape

The GPV experiment consisted of four plots (marked in green) with an area of 9 m × 9 m each for every treatment (T1 to T4, see below). Each plot contained three separate areas (3 m × 9 m) for (a) yield evaluation, (b) gas measurements, and (c) other samplings. In accordance with the requirements of the NH_3_ measurement method, all plots of GPV were surrounded by specially managed interspaces (9 m × 9 m, exemplified by a blue arrow in Fig. 1). This design allows a comprehensive evaluation of plant development, soil conditions and gaseous emissions. The experiments PVI and PVII made use of only one plot per treatment in order to evaluate the yield of the two other crops in the respective year.

The whole experiment was set up as a randomized design with four replicated plots and four treatments (T): (T1) Control - No N fertilization, (T2) Stabilised – double stabilised urea fertilization, (T3) Incorporated – subsurface placement, and (T4) Surface – granular urea surface application without UI + NI, without. All activities on the fields were conducted according to best agricultural management practices.

### Management

All management activities at each field plot were documented from late summer 2016 until late summer 2019. Mandatory data on management events were emergence, sowing, harvest with crop name, soil tillage with soil depth and type, applications of mineral and/or organic fertilization (including total amount of fertiliser and quantity of N-input from the fertiliser) as well as crop protection. Each activity and the associated device were described. Additionally, dates of crop development, damages as well as nutrition supply and previous crop were reported.

### Fertilisation

The amount of fertiliser applied was determined by the site-specific N requirement for each crop following the fertilisation recommendation of the associated Federal State (Saxony-Anhalt, Saxony and Bavaria); relevant details are summarized in Table 4. Three different N fertiliser treatments were tested: (T2) granular stabilised urea (ALZON® neo-N – combined use of urease and nitrification inhibitors (short: stabilised) also as surface application without incorporation. N-(2-nitrophenyl) phosphoric triamide (2-NPT)^26,27^ was used as urease inhibitor (UI) in the experiment, and the nitrification inhibitor (NI) was N-[3(5)-methyl-1H-pyrazol-1-yl) methyl] acetamide (MPA)^28^. (T3) subsurface placement is a special side dressing technology incorporating granular urea (PIAGRAN® 46) in combination with mechanic weed control (short: incorporated). This innovative technology was developed within the StaPlaRes project. (T4) granular urea surface application (PIAGRAN® 46) without incorporation (short: surface).

**Table 4:** Treatments and fertilization (Large plot experiment) (*before beginning of vegetation).

Fertilization treatment	Crop	Total N amount	Beginning of vegetation (VB)	Beginning of shooting (BBCH 32)	Until the end of shooting (BBCH 37/39)	Until the beginning of heading (BBCH 49/51)
		**BER/CUN/ROG**				
(T2) Stabilised	Winter oilseed rape	130/180/180 kg N ha^−1^	130/180/180 kg N ha^−1^*	—	—	—
(T3) Incorporated			130/100/60 kg N ha^−1^	–/80/120 kg N ha^−1^		—
(T4) Surface			130/100/60 kg N ha^−1^	–/ 80/120 kg N ha^−1^		—
(T2) Stabilised	Winter wheat	200/200/180 kg N ha^−1^	100/100/80 kg N ha^−1^	100/100/100 kg N ha^−1^		
(T3) Incorporated			70/70/40 kg N ha^−1^	70/130/60 kg N ha^−1^	60 /–/ 80 kg N ha^−1^	
(T4) Surface			70/70/40 kg N ha^−1^	70/70/60 kg N ha^−1^	60/60/80 kg N ha^−1^	
(T2) Stabilised	Winter barley	160/160/160 kg N ha^−1^	160 kg N ha^−1^	—	—	—
(T3) Incorporated			80/160/60 kg N ha^−1^	80/–/100 kg N ha^−1^		
(T4) Surface			80/80/60 kg N ha^−1^	80/80/100 kg N ha^−1^		—

For cereals, the first fertiliser application took place at the same time in all fertilised treatments. The number of split applications was reduced from three to two in winter wheat and from two to one in winter oilseed rape and winter barley for (T2) Stabilised. The stabilised one-time fertilisation for OSR was applied approx. two to three weeks earlier. The scheduling of the application of stabilised urea was studied with two fertiliser treatments: (a) granular stabilised urea (ALZON® neo-N – combined use of urease and nitrification inhibitors (short: stabilised) also as surface application without incorporation, (b) granular stabilised urea using ALZON® neo-N as a very early initial application (before the beginning of vegetation) and a flexible timing of the second dressing (shoot). An additional experiment was conducted in Cunnersdorf and Roggenstein for winter wheat and winter barley to optimise the timing of N-stabilised fertilisation (T2).

### Meteorological measurements

All meteorological parameters were measured in 60-minute resolution by different weather stations at each experimental site (see Table 5). The measurements included air humidity, air pressure, air temperature, global radiation, precipitation and wind speed.

**Table 5:** Meteorological parameters.

Meteorological parameters	Unit	Method(s)
Air humidity	%	gruuna meteo meter (BER & CUN), LfL weather station (ROG)
Air pressure	hPa	gruuna meteo meter (BER & CUN), LfL weather station (ROG)
Air temperature (200 cm height)	°C	gruuna meteo meter (BER & CUN), LfL weather station (ROG)
Global radiation	W/m²	gruuna meteo meter (BER & CUN), LfL weather station (ROG)
Precipitation	mm	gruuna meteo meter (hourly for BER & CUN), LfL weather station (hourly for ROG), official DWD weather station (daily for BER), farm weather station (daily for CUN),
Wind speed (20 cm & 200 cm height)	m/s	gruuna meteo meter (BER & CUN), LfL weather station (ROG)

### Crop field sampling

At the end of each cropping season, yield grain (all crops) and straw (for winter wheat and winter barley) were harvested on each field plot. All crop materials were weighed. Subsequently, quality parameters such as the nitrogen or crude protein content as well as dry matter content of all grain samples were determined. For winter oilseed rape, the oil content was also analysed. Furthermore, crop development parameters like BBCH, grains per ear, plants per m², etc. have been recorded. All crop parameters (quality and development) were determined by methods as specified in Table 6.

**Table 6:** Crop parameters.

Crop parameter (quality)	Site	Method
Weight of 1000 grains (TKM)	all	Weighing
Crude protein content	CUN & ROG	NIRS or calculated from grain N content
Dry matter content (grain)	all	NIRS
Grain N content	all	NIRS or calculated from crude protein content
Oil content	CUN & ROG	NIRS
Oil content	BER	Magnetic resonance spectroscopy (VDLUFA method handbook III 5.1.4)
Crude protein content	BER	Combustion (VDLUFA method handbook III 4.1.2)
Straw N content	ROG	Combustion (VDLUFA method handbook III 4.1.2)
Grain N content	CUN	Combustion (VDLUFA method handbook I A 2.2.5)
Straw N content	CUN	Combustion (elementar analysator Vario MAX CNS)
Grain N content & Straw N content	ROG	Combustion (elementar analysator Vario EL)
Straw N content	BER	Combustion (DIN-EN-ISO-16634-1)
**Crop parameter (development)**	**Site**	**Method**
Growing stages (BBCH)	CUN & ROG	Hack *et al*. 1992
Grains per ear	all	Counting
Plants per m²	all	Counting
Pods per plant	all	Counting
Stems per m²	all	Counting

### Soil field sampling

The topsoil (0–30 cm) was analysed at the beginning of the experiment. For each site, soil moisture data were collected hourly beside the field plots on a grass covered plot using SENTEK sensors based on the FDR methodology. The soil moisture was also directly measured during the Large plot experiment (GPV) in Cunnersdorf. Additionally, every month, soil samples were determined gravimetrically to calibrate the sensors. Soil samples were taken to determine NH_4_-N and NO_3_-N before the beginning of vegetation and after the harvest at 0–30 cm and 30–60 cm soil depth. After the first fertiliser application, mineral nitrogen in the soils was measured weekly and simultaneously with the gas flux measurements. Thus, with each gas flux measurement campaign, soil ammonium-N and soil nitrate-N content are related. All soil samples were stored at −20 °C until lab analysis (see Table 7).

**Table 7:** Analysis methods of soil mineral N.

Parameter	Site	Method
NH_4_-N & NO_3_-N & N_min_	BER	Extraction with 0.0125 M CaCl_2_ solution (VDLUFA method handbook I A 6.1.4.1)
NH_4_-N & NO_3_-N & N_min_	CUN	Extraction with 0.0125 M CaCl_2_ solution (VDLUFA method handbook I A 6.1.4.1)
NH_4_-N & NO_3_-N & N_min_	ROG	Extraction with 0.1 M KCL solution (VDLUFA method handbook I A 6.1.4.1)
Field capacity (usable)	BER	Drying with 105 °C and weighing
S_min_	BER	ICP-OES (VDLUFA method handbook I A 6.3.1)
S_min_	CUN	Extraction with 0.0125 M CaCl_2_ solution (VDLUFA method handbook I A 6.1.4.1)

### Crop and soil sampling of lab, pot and lysimeter experiments

In addition to the field experiments, process-related investigations were conducted. Under standardized laboratory conditions (20 °C) without plants, soil tests were applied to investigate effects of urea with or without inhibitors on the nitrogen turnover dynamic and urease activity. Furthermore, ammonia volatilization potential (AVP) was also tested under different temperature regimes (5 °C and 20 °C). All methodological details about AVP have been described by Ohnemus, *et al*.^29^. Several pot experiments with oat, silage maize, spring barley, spring wheat and summer oilseed rape using Mitscherlich containers were installed to analyse the nitrate leaching potential and/or ammonia volatilization potential. Lysimeter experiments served to quantify the amount of nitrate leaching for two fertiliser treatments (T2 and T3).

### Gas field measurements

The static closed chamber technique (modified based on^30–32^) was installed at all three sites to measure N_2_O, CO_2_ and CH_4_ during the crop cultivation period of winter oilseed rape, winter wheat and winter barley only for the “Large plot experiment” (see Fig. 1). Gaseous emissions were measured weekly and event-related in the morning until noon, i.e. weekly from the beginning after sowing and two times per week in loss-prone phases - wetness, fertilization, freeze-thaw. The chambers equipped with four sampling valves on the top were placed on chamber frames, which were installed in the ground shortly before the start of measurement and remained closed there for 60 minutes. The gas samples taken at twenty-minute intervals from the closed chambers were pumped out using 50 ml syringes and transferred to closed 20 ml crimp-top vials with rubber septa. In the end, four gas samples per plot were collected and analysed with a gas chromatograph. The field flux measurements and analysis of measurements have been described in detail by Vinzent, *et al*.^33^, Ruser, *et al*.^34^, Flessa, *et al*.^35^, Kesenheimer, *et al*.^13^. They were used at all experimental sites. At Bernburg and Cunnersdorf, N_2_O and CO_2_ were measured, while at Roggenstein CH_4_ was also analysed. There were differences of the chamber system (e.g. chamber area and chamber volume – both mentioned for each measurement) and the GHG flux calculation (details provided in Table 8 for the three field sites).

**Table 8:** Methods of GHG flux calculation.

Site	Flux calculation method	Available at
BER	R Package „flux“^47^	https://cran.r-project.org/web/packages/flux/flux.pdf
CUN	R Package „flux“^47^ revised by ZALF and adapted by SKW	https://cran.r-project.org/web/packages/flux/flux.pdf Adapted script is stored together with the StaPlaRes-DB-Thuenen
ROG	R Package „gasfluxes“^48^	https://cran.r-project.org/web/packages/gasfluxes/gasfluxes.pdf

#### Ammonia field measurements

Emissions of NH_3_ after fertilization were recorded using the method of Calibrated Passive Sampling - a combination of Dynamic Tube Method (DTM) and Passive Samplers^36^. The basic idea of this approach is to combine a simple qualitative measurement method on many field plots with a quantitative method with parallel measurements on a few plots. I.e. passive samplers^37^ filled with diluted sulphuric acid continuously absorb ammonia. DTM^38–40^ was applied in short measurement periods throughout the day. All details about the experimental design, operational instructions, preparations and flux calculation have been described with video instructions and material list by Pacholski^36^.

#### N_2_ flux determination

For each field site, soil samples were taken to conduct experiments under different boundary conditions (see Table 9) to measure and to analyse N_2_ and N_2_O flux in a fully automated system with the N_2_-free helium-oxygen incubation method. Previous N_2_ studies by Fiedler, *et al*.^41^, Butterbach-Bahl, *et al*.^42^, Buchen-Tschiskale, *et al*.^43^. outlined the principle of the investigation. The described procedure has been applied here for the first time.

**Table 9:** N_2_ flux determination experiments (^1^BER: 79%).

Experiment	Fertilization	aerial	WFPS
Exp1	Control (without any fertiliser)	aerobe	TR1: 70%/TR2: 90%^1^
Exp2	Control (without any fertiliser)	anaerobe	TR1: 70%/TR2: 90%
Exp3	TR3: Calcium chloride nitrate/ TR4: Urea	aerobe	70%
Exp4	TR3: Calcium chloride nitrate/ TR4: Urea	anaerobe	70%

This method includes three soil cores with a volume of 250 cm³ for the incubation and nine soil cores with a volume of 100 cm³ for N_min_-analyses. Analyses were conducted at the beginning of gas flux measurement (t_0_), at the peak of the N_2_O release (t_1_), at the peak of the N_2_ release (t_2_) and at the end of the gas flux measurement.

Dry soil and water were mixed to obtain a water filled pore space (WFPS) of 70% (TR1) and 90% (TR2) for experiment 1 and 2. For 2 days, the soil cores (250 cm³) were left at 20 °C. Subsequently, the soil cores and fertiliser solution were cooled down to 1 °C and then the fertiliser solution (TR3 and TR4) was injected with five punctures (250 cm³) and four punctures (100 cm³) by a hole template. Soil samples were placed in a helium incubation system and incubated at 1 °C. The normal air was removed from the system and replaced by a helium-oxygen mixture three times. The change in N_2_ concentration was measured for two to three days. When consistently low N_2_ values were reached, the helium-oxygen mixture was replaced by a more complex N_2_-free gas mixture (He/O_2_/trace gases). After that the temperature in the system was increased to 20 °C. The measurements of N_2_ and N_2_O were carried out up to two weeks until concentrations had levelled off again, i.e. the measured concentrations were similar to the level of the He/O_2_/trace gas mixture used for incubation. A detailed description of the preparation and incubation is stored with StaPlaRes-DB-Thuenen.

#### Modelling data

Soil moisture and seepage of each experimental site was modelled using the agricultural meteorological hydrologic budget model METVER. Meteorological and soil physical data as well as data on the crop phenological development is required for METVER. The meteorological data include daily mean air temperature, daily sunshine duration and daily precipitation. Further information about METVER is published by Böttcher, *et al*.^44^.

## Data Records

All data are stored in the relational database StaPlaRes-DB-Thuenen and are available on the publication server and data repository OpenAgrar (OA)^20^ (10.3220/DATA20220119144442). OA is the collective open access repository of research institutions affiliated with the Federal Ministry of Food and Agriculture (BMEL) in Germany. The open access repository publishes, stores, archives and distributes publications, publication references and research data. Its resources can be searched and used by everyone. It contains theses, reports, conference proceedings, journal articles, books, institutional documents, research datasets, videos and interviews. The repository is registered in re3data.org to improve data finding.

**Fig. 2:**
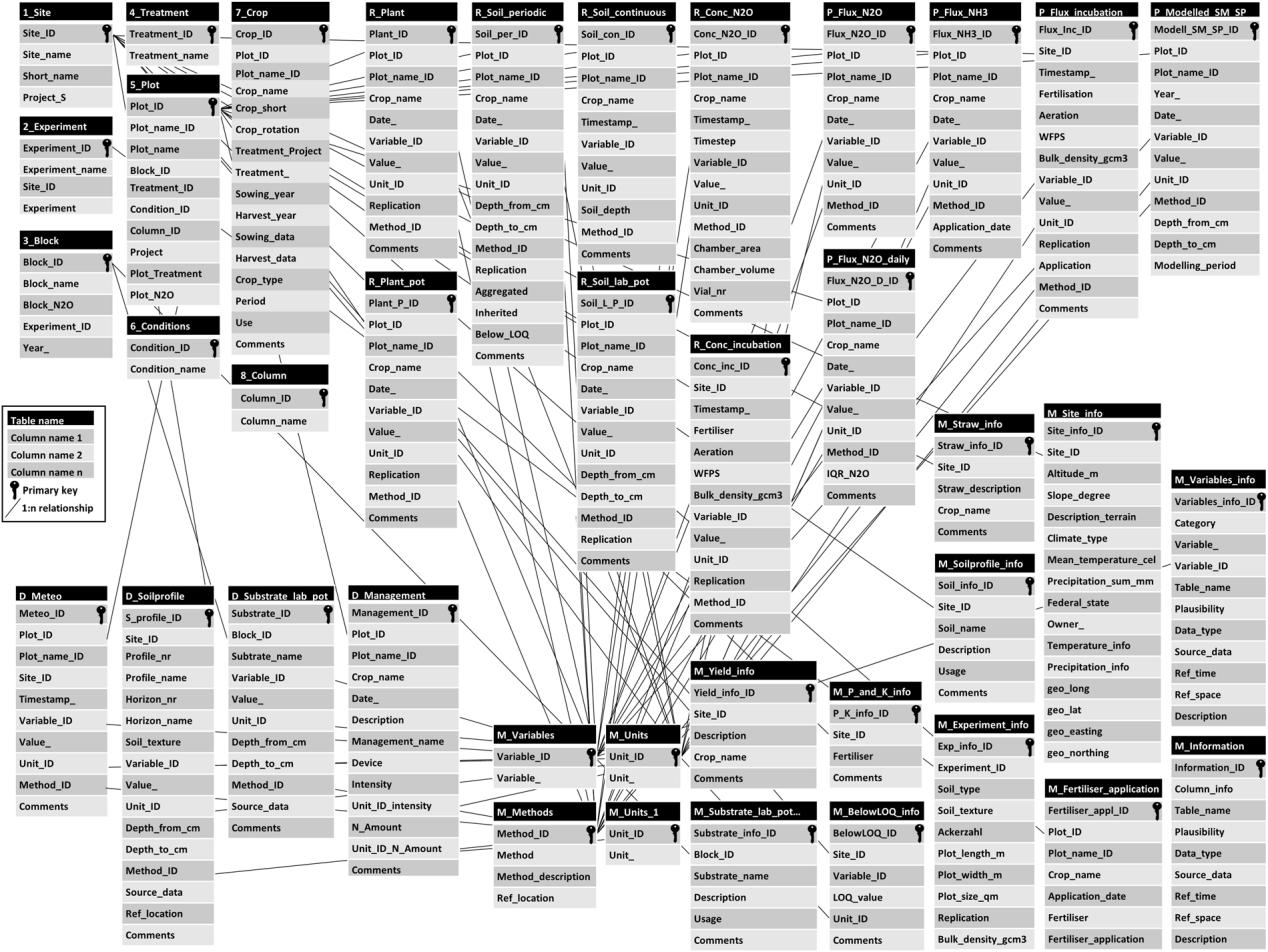
Data structure of the database StaPlaRes-DB-Thuenen.

StaPlaRes-DB-Thuenen has been designed with Microsoft Access 2019. The database provides stored and archived data (in total 2.5 million records) spread over 38 separate tables (see Table 10). The database tables are related to each other via primary and secondary keys. For simplification, all tables are organised in categories: “experimental design”, “driving forces”, “measurements – raw data”, “measurements - processed data”, “specific statistics” and “metadata”. Figure 2 shows the data structure of the database. More details about the database are provided in its documentation.

**Table 10:** All tables of the StaPlaRes-DB-Thuenen.

Table name	Category
D_Management	Driving forces
D_Meteo	Driving forces
D_Soilprofile	Driving forces
D_Substrate_lab_pot	Driving forces
1_Site	Experimental design
2_Experiment	Experimental design
3_Block	Experimental design
4_Treatment	Experimental design
5_Plot	Experimental design
6_Conditions	Experimental design
7_Crop	Experimental design
8_Column	Experimental design
M_Below_LOQ_info	Metadata
M_Experiment_info	Metadata
M_Fertiliser_application_info	Metadata
M_Information	Metadata
M_Methods	Metadata
M_P_and_K_info	Metadata
M_Site_info	Metadata
M_Soilprofile_info	Metadata
M_Substrate_lab_pot_info	Metadata
M_Straw_info	Metadata
M_Units	Metadata
M_Variables	Metadata
M_Variable_info	Metadata
M_Yield_info	Metadata
P_Flux_incubation	Processed data - Measurements
P_Flux_N2O	Processed data - Measurements
P_Flux_N2O_daily	Processed data - Measurements
P_Flux_NH3	Processed data - Measurements
P_Modelled_SM_SP	Processed data - Measurements
R_Conc_incubation	Raw data - Measurements
R_Conc_N2O	Raw data - Measurements
R_Plant	Raw data - Measurements
R_Plant_pot	Raw data - Measurements
R_Soil_continuous	Raw data - Measurements
R_Soil_periodic	Raw data - Measurements
R_Soil_lab_pot	Raw data - Measurements

### Category - EXPERIMENTAL DESIGN

The category “experimental design” contains the basic information (“key of the database”). The table “5_Plot” represents the organizing principle of the database and contains a Plot_ID (the primary key) describing the unique positioning or affiliation of each measured value and the associated information of the database. For each “Measurements” table in the StaPlaRes-DB-Thuenen there is a 1:n relation to the table “5_Plot”. This means that the tables are linked by the foreign key Plot_ID (with the exception of the tables “R_Conc_incubation” and “P_Flux_incubation”). These measurements-tables and the metadata-tables “M_Site_info”, “M_Straw_info”, “M_BelowLOQ_info”, “M_Yield_info”, “M_P_and_K_info”, “M_Soilprofile_info” and “D_Soil_profile” (Driving forces) are linked to the table “1_Site” via the Site_ID as a foreign key.

### Category – DRIVING FORCES

A dataset in the table “D_Management” describes what event or what activity (Management_Name) was performed on a specific plot for a particular crop, at a given time (as date) with a certain intensity (Intensity), the used device and the amount of N in case of nitrogen fertilisation. The columns Intensity and N_amount are complemented by a unit as index.

“D_Soil_profile” describes the composition of the soil profile at each site (location) consisting of horizons (Horizont_nr, Horizont_name) and relevant parameters (soil texture, measured value, unit as index, soil depth from, soil depth to, method as index, source of data, comment). All meteorological parameters, displayed in Table 5, are stored in the table “D_Meteo”.

### Category – MEASUREMENTS

All “measurements” tables are structured with the following eight columns. If necessary each table can be complemented by more columns.

**Table:** 

Plot_ID:	Unique spatial positioning/affiliation of the measured value
Date_ or Timestamp_:	Point in time of the measured value as date (dd.mm.yyyy) or timestamp (dd.mm.yyyy hh:mm:ss)
Variable_ID:	Index of the measured variable
Value_:	The measured value
Unit_ID:	Index of the unit in which the measured value was recorded
Method_ID:	Index of the applied methods of the measured value
Comments:	Comment(s)
ID:	Unique counter/index of the table

Column names are sometimes underlined at the end because they differ from the reserved words in the Access database and to avoid problems/error messages. Reserved words are words and symbols with a special meaning for Microsoft Access. The metadata tables “M_Variables”, “M_Units” and “M_Methods” are always linked to each “measurements” table. Please note that not all measurements are available across all field sites.

### Data of crop and soil samplings

The tables “R_Plant” and “R_Soil_periodic” contain all event-related plant and soil field samples. “R_Soil_periodic” is additionally equipped with “soil depth from” and “soil depth to” as well as with three Boolean columns (switching variable). “Aggregated” column indicates whether a measured value was aggregated based on several values or not. Whether a measured value was adopted from another plot or not will be shown by “Inherited” as a second Boolean column (if a value was adopted, a comment indicates from which plot). A further Boolean column “Below_LOQ” in this table indicates whether a measured value is below the limit of quantification (LOQ) or not. “R_Soil_continuous” stores all soil sensor values which were measured in an hourly interval. In addition to field samples, laboratory samples for soil tests and pot experiments were conducted. For a clear differentiation of the different “scale” of measurements, all lab or pot measured values are stored in the table “R_soil_lab_pot” and “R_Plant_pot”.

### Gas emission data

The database contains raw data of gas flux measurements (table “R_Conc”) and processed data (“P_N2O_flux” and “P_NH3_flux”). Table “R_conc” lists the specific concentration of the gases measured by gas chromatography and used for the calculation of the respective gas fluxes. table is supplemented by the columns “time step”, “chamber area”, “chamber volume” and “vial number”. The gas fluxes of N_2_O, CH_4_ and CO_2_ are stored in the table “P_N2O_flux”. “P_N2O_flux_daily” provides interpolated and aggregated daily N_2_O fluxes. “P_NH3_flux” table contains NH_3_ fluxes.

### Additional gas data and modelling data

In additional laboratory experiments concentrations and fluxes of N_2_, N_2_O, CO_2_ and CH_4_ were quantified using the described incubation method. The experimental results are displayed in the table “R_Conc_incubation” and “P_Flux_incubation”. Modelled values of soil moisture and seepage are stored in the database table “P_Modelled_SM_SP”.

### Category – METADATA

All variables, units and methods used in the StaPlaRes-DB-Thuenen are listed in the metadata tables “M_Variables”, “M_Units” and “M_Methods”. “M_Variables_info” displays all variables used. The information about variables contains a brief description and is supplemented by value plausibility and reference to time and space. The data type of each variable is also defined (raw data, processed or general data). The table “M_Information” defines descriptive information on all columns of the StaPlaRes-DB-Thuenen, except for the column “Variable_“. All table names include “info” at the end. Further metadata tables provide additional information; which is described below.

- sites - All field sites are described with general information about the site, such as coordinates, altitude above NN, slope, climate type (USDA Plant Hardiness Zones), mean annual temperature, etc.
- experiments – Field experiments are described by general information about the experiment, such as soil type, soil texture, plot size, etc.
- crop yield – Residual moisture content of the grain yield for the investigated crops.
- straw yield – the handling of straw after the harvest (whether the straw was incorporated or removed from the field).
- fertiliser application – Due to weather conditions, it was not always possible to apply the urea with subsurface placement. Plot by plot it is described on which fertilisation date the fertilisation was “incorporated” or defined as “surface” fertiliser application. If the table does not contain an entry there was no deviation in fertiliser application from the treatment.
- phosphor & potassium fertilization – the handling of phosphor and potassium fertiliser application at each field site (stock or annual fertilization).
- limits of quantification – for different measured variables (e.g. NH4-N) limits of quantification (LOQ) are documented.

## Technical Validation

**Fig. 3:**
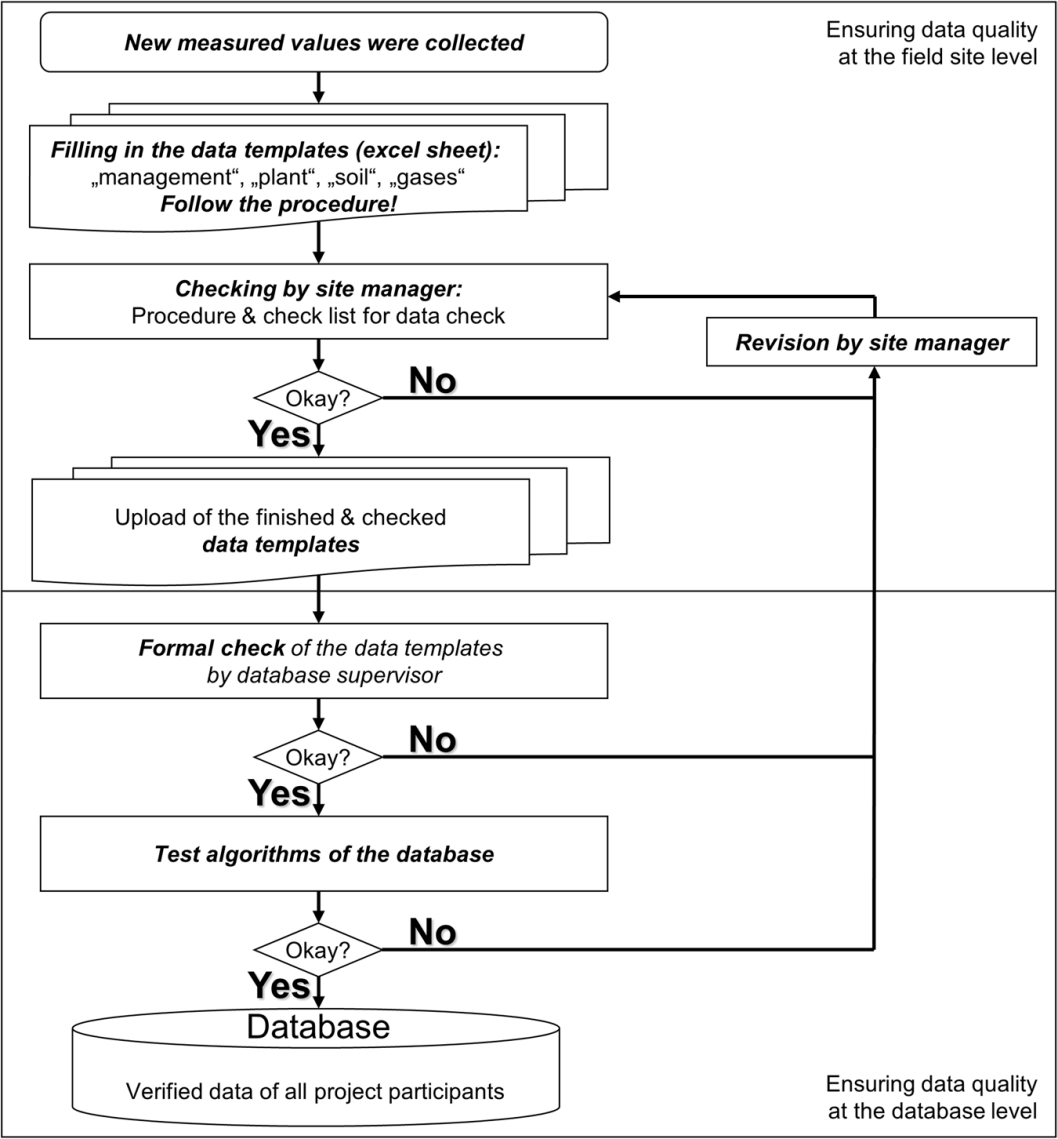
Flow chart of data quality management.

Data have been collected by tailored data templates which have been compiled in an iterative manner. By pre-defining experiment names, treatment names, measurement variables, units and methods in the data templates, it was possible to reduce errors. In addition, a two-level data quality control was elaborated. The flow chart in Fig. 3 illustrates the procedure.

A document was provided with detailed explanations on the procedure for data delivery and data quality control. Moreover, we plotted each variable to identify and correct errors in data entry, as well as to identify and remove potential erroneous measurements.

Multiple steps were taken to ensure the technical quality of the dataset. Most importantly, consistent field and laboratory protocols were employed. For example, ring tests for gas chromatograph analysis of N_2_O have been conducted at all laboratory.

The results from helium incubations were specifically checked for the following measurement errors: erratic, synchronous changes in all gas concentrations, measurement gaps, negative CO_2_ and N_2_ concentration values, deviations between the measured and expected concentration values for internal gas standards.

Crop yield data were compared to yield from other experiments conducted at the same location or to yield data from national variety trails. A one-way ANOVA was conducted to compare the mean crop yield of each treatment. A Tukey’s post-hoc test was performed for a pairwise comparison of the treatments with a statistical difference at p < 0.05.

Due to irregularities in the hourly precipitation data from the measurement technology at the CUN and BER sites, daily precipitation data from parallel existing weather stations were supplemented (see Table 5).

## Usage Notes

The data described are stored in the database StaPlaRes-DB-Thuenen and will be freely available for (re)use by others at the publication server and data repository OpenAgrar^20^ (10.3220/DATA20220119144442).

Database protection and data reproducibility was guaranteed by dividing the StaPlaRes-DB-Thuenen into a frontend database (FE) and a backend database (BE). The frontend (labelled with “fe” in the database name) represents the application database. The backend (labelled with “be” in the database name) embodies the base of the data in the background and is not intended for application. StaPlaRes-DB-Thuenen has been developed in Microsoft Access 2019 and tested for program version 2016, 2019 and 365. The download of the database contains detailed instructions, which describe how to open and use the StaPlaRes-DB-Thuenen.

## Data Availability

The code used for the N_2_O flux calculation is open-source and was published in the Comprehensive R Archive Network (CRAN). Each used script is mentioned in Table 8. All codes used to calculate NH_3_ fluxes are stored together with the StaPlaRes-DB-Thuenen on the repository OA^20^. The model METVER was developed by Böttcher, *et al*.^44^ from the German Weather Service (DWD). The model source code is published by Bach^45^ (see Appendix) and can be downloaded from OA repository^20^ as well.
